# Plant-Herbivore Interaction: Dissection of the Cellular Pattern of *Tetranychus urticae* Feeding on the Host Plant

**DOI:** 10.3389/fpls.2016.01105

**Published:** 2016-07-27

**Authors:** Nicolas Bensoussan, M. Estrella Santamaria, Vladimir Zhurov, Isabel Diaz, Miodrag Grbić, Vojislava Grbić

**Affiliations:** ^1^Department of Biology, The University of Western OntarioLondon, ON, Canada; ^2^Centro de Biotecnologia y Genomica de Plantas, Instituto Nacional de Investigacion y Tecnologia Agraria y Alimentaria, Universidad Politecnica de MadridMadrid, Spain; ^3^Departamento de Agricultura y Alimentacion, Universidad de La RiojaLogrono, Spain

**Keywords:** *Tetranychus urticae*, two-spotted spider mite, plant-pest interaction, stylet, chlorosis, microscopy, bean, *Arabidopsis*

## Abstract

The two-spotted spider mite, *Tetranychus urticae* Koch (Acari: Tetranychidae), is one of the most polyphagous herbivores feeding on cell contents of over 1100 plant species including more than 150 crops. It is being established as a model for chelicerate herbivores with tools that enable tracking of reciprocal responses in plant-spider mite interactions. However, despite their important pest status and a growing understanding of the molecular basis of interactions with plant hosts, knowledge of the way mites interface with the plant while feeding and the plant damage directly inflicted by mites is lacking. Here, utilizing histology and microscopy methods, we uncovered several key features of *T. urticae* feeding. By following the stylet path within the plant tissue, we determined that the stylet penetrates the leaf either in between epidermal pavement cells or through a stomatal opening, without damaging the epidermal cellular layer. Our recordings of mite feeding established that duration of the feeding event ranges from several minutes to more than half an hour, during which time mites consume a single mesophyll cell in a pattern that is common to both bean and *Arabidopsis* plant hosts. In addition, this study determined that leaf chlorotic spots, a common symptom of mite herbivory, do not form as an immediate consequence of mite feeding. Our results establish a cellular context for the plant-spider mite interaction that will support our understanding of the molecular mechanisms and cell signaling associated with spider mite feeding.

## Introduction

The chelicerates are the second largest arthropod group comprising of horseshoe crabs, scorpions, spiders, mites, and ticks (Brusca and Brusca, 2003). Horseshoe crabs, scorpions and spiders are predators that use pre-oral digestion as a shared digestive strategy. These organisms secret digestive enzymes, originating from the midgut, into their prey to aid in the pre-oral digestion and liquefaction of prey tissues before ingestion by morphologically diverse mouthparts (Cohen, 1995). Ticks and mites belong to Acari, the most diverse group within chelicerates, with over 40,000 identified species. This group exhibits a plethora of different lifestyles ranging from parasitic to predatory to plant-feeding. Predatory Acari, similar to other chelicerate predators, utilize secreted proteins to facilitate the consumption of their prey. However, these digestive enzymes originate from salivary secretions rather than from the midgut (Cohen, 1995). Phytophagous mites represent a complex assemblage of saprophagous, fungivorous and herbivorous species (Krantz and Lindquist, 1979). Among them, Tetranychidae (spider mites), Tenuipalpidae (false spider mites), and some Eriophyoidea mites are exclusively phytophagous and include major agricultural pests. A common feature of the mouth apparatus of these mites includes the formation of the elongated cheliceral stylet that allowed adaptation to a piercing mode of feeding, in which the stylet is used to penetrate the host tissue to allow the consumption of the cell content. While Tetranychidae and Tenuipalpidae mites have a single, long and retractable stylet (Alberti and Kitajima, 2014), Eriophyoid mites have several stylets that are not retractable. Instead, Eriophyoid mites penetrate their stylets into the plant tissue by telescoping the palpal tissue that surrounds the stylet bundle (Krantz and Lindquist, 1979).

*Tetranychus urticae* Koch (Acari: Tetranychidae), the two-spotted spider mite (TSSM), is one of the most polyphagous herbivores that feeds on over 1100 plant species, including more than 150 crops species (Jeppson et al., 1975; Migeon and Dorkeld, 2006–2016). Similarly to phloem-feeding insects, this chelicerate pest has mouthparts adapted for a sucking mode of feeding, but exactly how *T. urticae* feeds on plant tissues remains controversial. In contrast to phloem-feeding herbivores that suck the sap from a plant's vascular system, TSSMs feed on cells within the leaf mesophyll (Park and Lee, 2002). Associated with this difference in feeding preference is the length of the TSSM stylet that ranges from 100μm in larvae to ~150 μm in adult female mites (Avery and Briggs, 1968; Ekka, 1969; Sances et al., 1979), relative to the much longer stylets of phloem-feeding insects that can reach up to 800μm (Pointeau et al., 2012). The TSSM stylet is a tube formed by the interlocking of two cheliceral digits with a single canal of ~2μm in diameter (Andre and Remacle, 1984). This contrasts the more elaborate structure of stylets in phloem-feeding insects (e.g., aphids and psyllids), which consist of two canals: a feeding canal that transports the plant nutritive sap, and the salivary canal that allows secretion from the insect's salivary glands into the plant tissue (Tjallingii and Esch, 1993; Garzo et al., 2012). While the function of a stylet as a piercing-feeding organ is clearly described in phloem-feeding insects, its role in TSSM feeding is still unclear. It is not known if TSSM use their stylet to transport both the saliva and the plant nutritive fluid (Summers et al., 1973; Hislop and Jeppson, 1976; Andre and Remacle, 1984), or if they use the stylet to pierce the plant tissues, deliver salivary secretions, and then use the buccal cavity to directly ingest the nutritive fluid originating from mesophyll cells that is proposed to be extruded to the surface by putative capillary action (Alberti and Crooker, 1985; Nuzzaci and De Lillo, 1991a).

Spider mites most frequently feed on leaf tissues, causing the formation of chlorotic spots that are associated with an extensive collapse of the mesophyll layer (Sances et al., 1979; Park and Lee, 2002). Ultrastructural studies of damaged plant tissue identified cells that were either plasmolysed, empty or collapsed, or had coagulated contents (Tanigoshi and Davis, 1978; Albrigo et al., 1981; Campbell et al., 1990). As cell wall disruption was associated with some of the affected cells, the observed damage was attributed to stylet penetration and mite feeding. In addition, it has been estimated that mites damage ~20 clustered cells per minute directly leading to the formation of a chlorotic spot (Liesering, 1960). While these studies provide a benchmark for our understanding of the TSSM-plant relationship, some conclusions were inferred from the observation of the TSSM feeding behavior or its long-term consequence on plant tissues, rather than on the direct and immediate analysis of plant-mite interface. For example, the assessment of plant damage was based on the analysis of leaf tissues that were exposed to mite herbivory for days, hindering the ability to distinguish between plant damage that directly resulted from mite feeding and damage that was a cumulative result of both mite feeding and plant's response to it. In addition, the number of cells consumed by mites was estimated based on the movement of TSSM's mandibular plate and the assumption that every movement corresponds to the insertion of the stylet into an independent cell (Liesering, 1960).

*T. urticae* is being established as a model chelicerate pest. Its genome was recently sequenced (Grbic et al., 2011) and several plant-TSSM interaction experimental systems were established (Zhurov et al., 2014; Martel et al., 2015; Wybouw et al., 2015; Diaz-Riquelme et al., 2016). The ability to track the whole-genome reciprocal responses in plant-TSSM interactions allows dissection of molecular mechanisms underlying plant responses to mite herbivory. In addition, recent work indicated that the TSSM and related mites can manipulate plant defense responses, suggesting that there is an elaborate interaction between these herbivores and their plant hosts, and an evolutionary arms-race between their genomes (Kant et al., 2008; Alba et al., 2014; Wybouw et al., 2015; Villarroel et al., 2016). In this context, the knowledge of spider mite feeding at the cellular level, using direct observations, becomes critical for understanding the cell interactions and signaling underlying mite feeding and plant-induced responses. Here, we characterized plant damage that directly results from mite feeding at a cellular level and the interface between the mite and the plant during the feeding process. We show that TSSM uses its stylet to penetrate into the leaf mesophyll, where it consumes individual cells without damaging epidermal cell layer. We show that the consumption rate of plant cells is much lower than previously estimated and that mite feeding *per se* does not result in the formation of chlorotic spots. This study establishes the cellular context for the plant-spider mite interactions required for our understanding of the cell signaling associated with spider mite feeding.

## Materials and methods

### Plant growth and mite rearing

The bean, *Phaseolus vulgaris*, cultivar California Red Kidney (Stokes, Thorold, ON), and *Arabidopsis thaliana* (Columbia-0) plants were grown from seed in peat–vermiculite growing mix (Premier Pro-mix BX; Premier Tech) at 24°C, under 100–150μM m^−2^s^−1^ cool-white fluorescent light and 16/8 h (light/dark) photoperiod. The reference spider mite strain, *Tetranychus urticae* (London), was reared on bean plants under same conditions.

### Monitoring of *T. urticae* feeding

To estimate the amount of time that spider mites spend at a feeding site, mites were first starved for 12 h and then 10 female mites were placed on either bean or *Arabidopsis* leaf disk of 1.5 cm in diameter. Mite feeding was recorded under a dissecting microscope fitted with the Canon EOS Rebel T5i camera (Canon, Japan).

### *T. urticae* cuticular preparations

Adult spider mites were starved for 12 h and were placed in 100% ethanol overnight. The following day, mites were transferred to a slide containing a drop of a mix of Hoyer's medium and lactic acid (1:1, v/v). The slides were incubated overnight at 60°C and were viewed using a Zeiss Axiophot microscope (Carl Zeiss AG, Germany) fitted with a Zeiss AxioCam Color HRc CCD Camera 412-312 (Carl Zeiss AG, Germany).

### Phalloidin staining

Spider mite adults were collected and fixed in 4% formaldehyde in 0.1 M phosphate buffer saline (pH 7.4) overnight at 4°C. Mites were washed twice in 0.1 M phosphate buffer. Approximately 100 spider mites suspended in 100μl of phosphate buffer saline were incubated with 50μl of phalloindin 546 (Alexa Fluor® 546 Phalloidin, ThermoFisher Scientific, USA) overnight at 4°C (Jiang et al., 2007). Phalloidin stained actin filaments were visualized using a Zeiss Axiophot confocal microscope (Carl Zeiss AG, Germany) using the following settings: Excitation: 543 nm, Filter: Ch1, LP 560; Image size: 2048 x 2048 pixels representing an area of 460.6 × 460.6μm.

### Scanning electron microscopy

Spider mites were fixed overnight in 2.5% (v/w) glutaraldehyde (Electron Microscopy Sciences, USA) in 0.1 M sodium phosphate buffer (pH 7.2). Fixed mites were washed three times in 0.1 M sodium phosphate buffer (pH 7.2) and were dehydrated in a graded ethanol series of increasing concentration for 10 min at each concentration—50, 70, 80, 90, 95, 100%, and 100% (v/v in H_2_O). The specimens were further dried in a graded series of hexamethyldisilazane (HMDS; Sigma, USA) diluted with 100% ethanol (v/v) to 25, 50, 80, 100, 100% for 10 min at each step, followed by air drying in a fume hood for 1 h (Bray et al., 1993). Individual spider mites were mounted onto SEM stubs using an eyelash probe before coating them with gold particles in the sputter (Technics Hummer VI Sputter Coat Unit, Anatech, USA). Samples were examined with a Hitachi S-3400N electron microscope (Hitachi Science Systems, Tokyo, Japan) operated at a voltage of 5 kV.

### Trypan blue staining of plant tissue and quantification of damage caused by *T. urticae* feeding

Trypan blue staining was performed according to Keogh et al. (1980), with some modifications. Adult female mites were allowed to feed on either the adaxial or abaxial side of the *Arabidopsis* or bean leaf piece of 1 cm^2^ for 10 min, as described above. Subsequently, leaves were submerged in trypan blue solution (1:1:1:1 v/v, lactic acid, phenol, glycerol, water, and 1% trypan blue) diluted with 95% ethanol (1:2 v/v) in a 15 mL conical polypropylene tube, and were placed in a boiling water bath. *Arabidopsis* leaves were boiled for a minute and bean leaves for 5 min. The tissue was left in the staining solution overnight at room temperature. Subsequently, leaves were cleared with chloral hydrate solution (2.5 g/mL diluted in water; Sigma, USA) for ~6 h with two changes of the solution. Cleared leaves were either prepared for imaging or sectioning. Leaves for *imaging* were mounted in 50% glycerol in 0.1 M sodium phosphate buffer at pH 7.0 and observed by light or confocal microscopy using a Zeiss Axiophot microscope (Carl Zeiss AG, Germany) fitted with a Carl Zeiss AxioCam Color HRc CCD Camera 412-312 (Carl Zeiss AG, Germany). For confocal imaging, the tissue was excited with a 543 nm HeNe laser line and bi-directional scanning was used to scan leaf tissue regions in sections of 230 × 230μm at a 2048 × 2048 pixel resolution. *Sectioning of the trypan blue stained and cleared plant tissues:* Trypan blue-stained bean tissue was prepared for sectioning by fixing the sample in 10% glutaraldehyde solution in 0.1 M sodium phosphate buffer (pH 7.0) overnight. Leaf tissue was manually dehydrated in an ethanol series up to 70% (v/v in H_2_O). Further dehydration and paraffin embedding was performed in tissue processor (Leica ASP300TP). Embedded leaf tissue was sectioned on a microtome (Leica RM2255 Microtome) at a thickness of 10μm. Sections were dewaxed in two 10 min changes of 100% xylene, were mounted with Permount^TM^ Mounting Medium (Fisher, USA) and were examined under a Carl Zeiss AxioCam Color HRc CCD Camera 412-312. Several attempts to paraffin-embed trypan blue stained *Arabidopsis* leaves resulted in an excessive tissue disruption. Instead, a free-hand sectioning of *Arabidopsis* tissue was performed. Briefly, a surgical blade (Feather, No. 23) was used to cut leaf sections of 100–150μm in thickness under dissecting microscope. Leaf sections were mounted flat between layers of 2% agar (w/v in H_2_O) to provide physical support for the leaf tissue. The agar embedded tissue was excised, mounted in 50% glycerol (v/v in H_2_O) and observed under a light microscope using a Zeiss Axiophot microscope (Carl Zeiss AG, Germany) fitted with a Carl Zeiss AxioCam Color HRc CCD Camera 412-312 (Carl Zeiss AG, Germany).

### Histological analysis of *T. urticae* stylet path through the plant tissue

Fully expanded adult bean or *Arabidopsis* leaves were cut into small pieces (0.4 × 0.8 cm) that were placed on wet cotton with either adaxial or abaxial sides exposed and then infested with 50 mites that were starved for 12 h. Mites were allowed to feed for 10 min. Next, leaf pieces (with mites still feeding on them) were submerged in liquid nitrogen in order to fix mites in their natural feeding position. Frozen leaf pieces were transferred to a solution of 2.5% (v/v) glutaraldehyde in 0.1 M sodium phosphate buffer at pH 7. After 24 h, the tissues were gently washed in 0.1 M sodium phosphate buffer at pH 7 and dehydrated in a graded alcohol series: 25, 50, 75, 95, 100% and 100% (v/v in H_2_O), for 15 min in each solution. Leaf pieces with mites still attached were embedded in LR White resin (Electron Microscopy Science, USA) and were cured overnight at 55°C. Specimens were cut with a Reichert Ultracut S ultramicrotome (Leica, Austria) into 1μm serial sections using a glass knife. Sections were stained with toluidine blue, 0.5% (w/v) in 0.1% (w/v) Na_2_CO_3_ in water, for 5 min on a slide warmer at 60°C and dried overnight at room temperature. Cross sections of mite-free plant leaves were prepared in parallel as controls.

## Results

### Determination of the *T. urticae* feeding event

**Figure 1:**
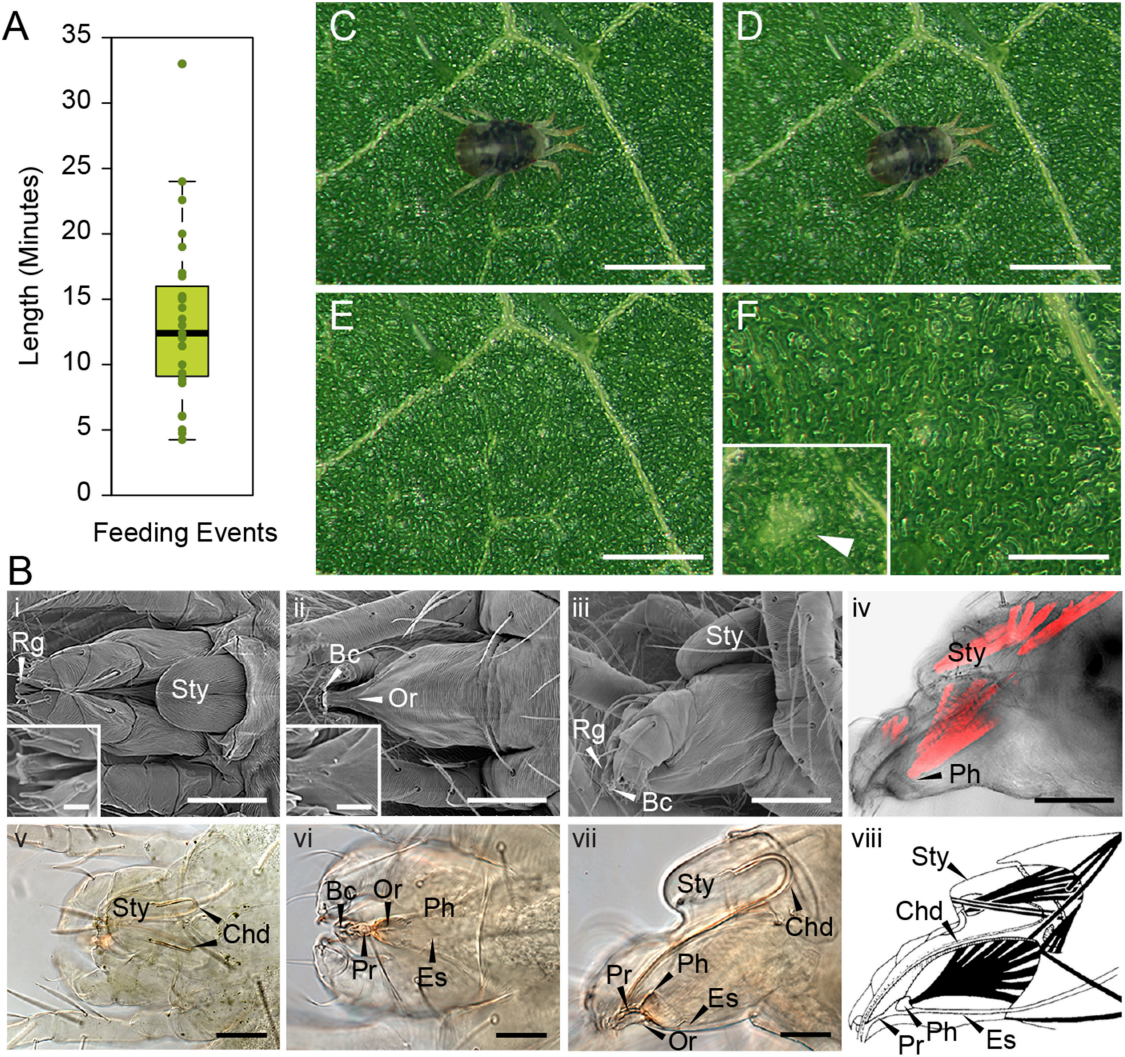
*****T. urticae*** feeding: duration and plant damage**. **(A)**, Duration of individual mite feeding events in minutes (*n* = 27). **(B)**, *T. urticae* mouth parts. i–iii, SEM imaging and v–vii, brightfield of mite gnathosoma. i and v, dorsal view with inset close up view of the rostral gutter (Rg); ii and vi, ventral view with inset close up view of the inferior oral commissure (Or); iii and vii lateral view; iv: phalloidin staining of actin filaments and viii: schematic representation of muscles associated with the mouth parts. Sty, stylophore; Bc, buccal cavity; Es, Esophagus; Ph, Pharynx; Pr, Propharynx; scale bars: in inset i and ii, 10μm; in iv (i–iv and vi–viii), 50μm; in v, 50μm. **(C–F)**, Mite feeding and plant damage. **(C)**, Mite at the beginning of the feeding event. **(D)**, Mite at the end of the feeding event. **(E,F)**, Feeding site. F inset, a typical chlorotic spot (arrow head). Scale bars: 500μm in **(C–E)**; 250μm in **(F)** (main panel and inset).

In order to determine the immediate consequence of mite herbivory on plant damage, we first had to establish the timing of mite's feeding event. A feeding event was defined as a process that is initiated by a mite's settlement at a particular leaf spot and is terminated when the mite raises its head away from the leaf surface. An example of a feeding initiation event is shown in the Supplemental Movie S1, beginning at 0 min 31 s, while a termination event can be seen at 5 min 2 s in the Supplemental Movie S2. The whole feeding event of this mite is shown in Supplemental Movie S3, which captures the mite feeding for over 12 min. In addition, two other mites that were continuously feeding are captured within the same frame. An analysis of 27 independent feeding events revealed a wide distribution of durations, ranging from as short as several minutes to over half an hour, with an average duration of mite feeding event of 13 min 22 s (Figure 1A).

During feeding, mites move their stylophore (Sty in Figure 1B) leading to stylet protrusion and penetration into the plant tissue. The stylet, not directly visible as mites feed, is a tube composed of two movable cheliceral digits (Chd in Figures 1Bv–viii). Each cheliceral digit is attached dorsally to a retractable and extrudable stylophore. While feeding, protracted cheliceral digits slide into groves formed by the rostral gutter (Rg; seen in the inset in Figure 1Bi) to interlock together and form the stylet—a hollow tube—at the buccal cavity level (Bc in Figure 1B). Besides the stylet and buccal cavity, the spider mite feeding apparatus is composed of the propharynx (Pr), pharynx (Ph), esophagus (Es), and midgut (Figure 1B). Ventrally, a small opening through the cuticle, referred to as the inferior oral commissure (Or) can be observed (see inset in Figure 1Bii). This aperture was hypothesized to help the suction flow caused by the pharyngeal pump (Nuzzaci and De Lillo, 1991b). Both the stylophore and the pharynx are connected to head muscles that control their movements (Figures 1Biv,viii). While the movement of the pharyngeal pump cannot be observed, the pulsing of the mite opistosoma (the posterior part of the body) is readily visible as mites feed (Supplemental Movies 1–3).

No visual plant damage can be observed following mite feeding. A representative feeding event is shown in Figures 1C,D. Once feeding is completed (this particular one lasted for 10 min), no macroscopic change can be observed at the site (Figures 1E,F). Thus, chlorotic spots that eventually form on infested leaves (see Figure 1F in inset) are not an immediate consequence of mite feeding. A similar duration of feeding with a lack of visible damage was also observed after mite feeding on the *Arabidopsis* leaves (data not shown). Thus, mites spend minutes feeding at the same leaf spot, without causing visual damage to plant tissue.

### Determination of *T. urticae* feeding pattern on plant tissue

The frequency of stylophore movement associated with mite feeding led to the proposition that mites consume ~20 plant cells per minute (Liesering, 1960). To determine the extent of leaf damage occurring during a single mite feeding event, we allowed mites to feed on leaves for 10 min, after which we immediately stained the leaf tissue with a vital stain—trypan blue—to identify the number and the pattern of dead cells. As host-plant preference may affect mite feeding patterns, we infested both bean leaves (preferred host for the population used in this study) and *Arabidopsis* leaves (a non-preferred host) with mites. In addition, since mites normally feed from both adaxial and abaxial leaf surfaces, we examined plant damage in a controlled experimental set-up that allowed mites to exclusively feed from only one of these surfaces. This allowed us to determine if accessing the leaf tissue from different epidermal surfaces has an effect on mite feeding patterns.

**Figure 2:**
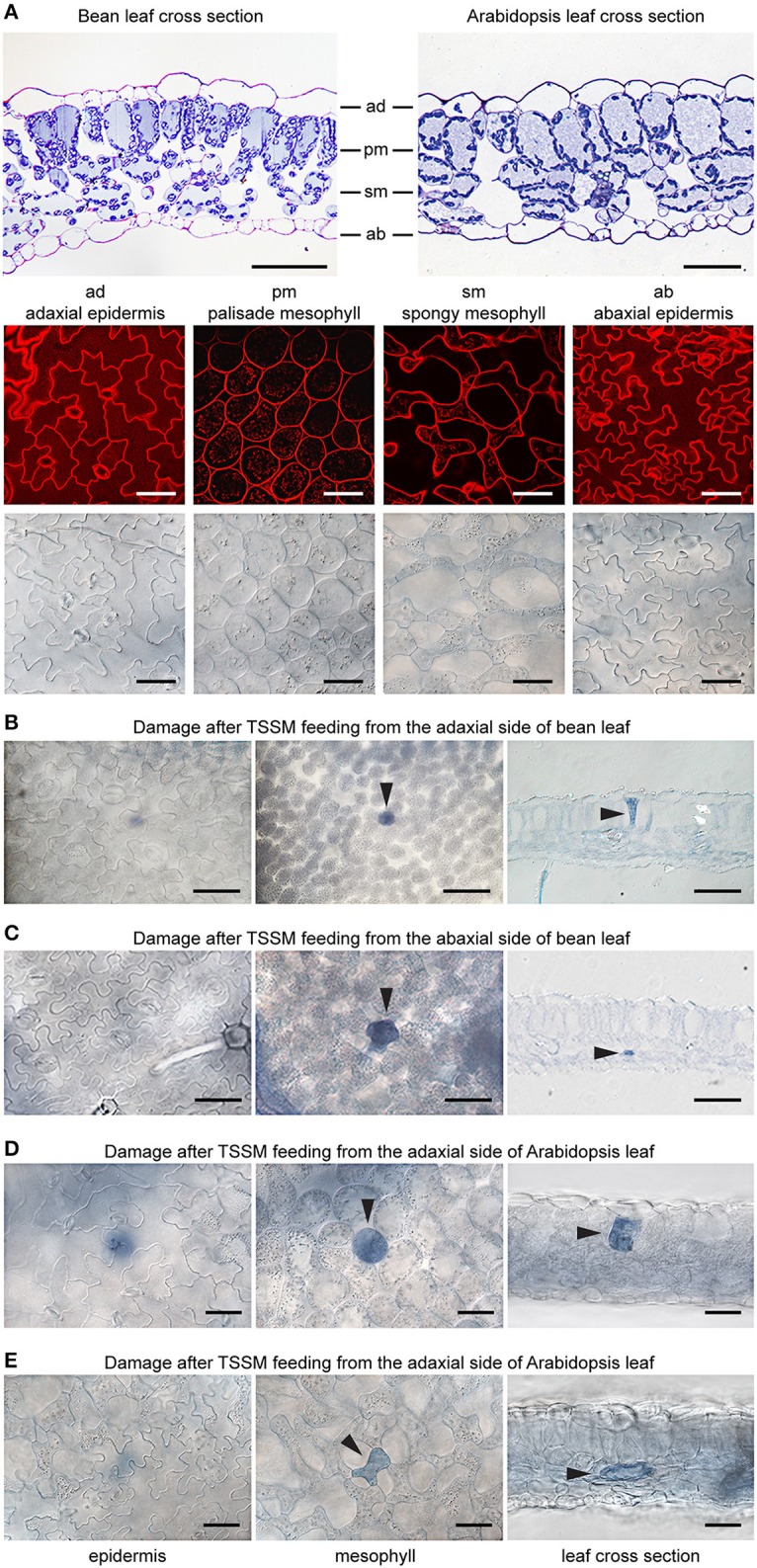
**Plant damage associated with spider mite feeding**. **(A)**, Longitudinal cross sections of bean and *Arabidopsis* leaves stained with toluidine blue (on the top) and *Arabidopsis* optical sections (at the bottom) visualized using confocal (upper row) and brightfield (lower row) microscopy. ad, adaxial epidermis; pm, palisade mesophyll; sm, spongy mesophyll; ab, abaxial epidermis. **(B–E)**, Representative images of damaged cells within trypan blue stained bean and *Arabidopsis* leaves after spider mite feeding for 10 min. Damaged cells appear blue and are marked with arrowheads in optical and cross sections. Scale bars: 50μm in **(A)** through **(E)**.

Bean and *Arabidopsis* leaves have cell layers typical of dicotyledonous plants: the adaxial (upper) epidermis (ad), the palisade mesophyll (pm), the spongy mesophyll (sm), and the abaxial (lower) epidermis (ab) (Figure 2A). We used both histological and optical sectioning to observe cells that were damaged as a result of mite feeding, Figure 2A. The epidermal layers are cellular monolayers containing densely packed pavement cells, trichomes, and stomata. Stomata consist of two guard cells that form an opening through the epidermal layers that allow gas exchange between the leaf mesophyll and atmosphere. Below the adaxial epidermis is the palisade mesophyll, a monolayer in both bean and *Arabidopsis* leaves that is composed of densely packed cylindrical cells. Located further ventrally is the spongy mesophyll that is characterized by a multilayer of oval and sparsely packed cells that are interspaced with large volumes of the air space. The spongy mesophyll is in direct contact with the abaxial epidermis, which forms the most ventral leaf boundary. We used trypan blue to distinguish intact cells from those with disrupted cell membranes, since only damaged cells accumulate the dye and appear blue under a bright field microscope. To ensure that we visualized all cells that could have been damaged during a 10-min mite feeding, we examined the leaf tissue in both transverse and longitudinal serial sections after mite feeding (Figures 2B–E).

When mites fed from the adaxial epidermis, the trypan blue staining was most frequently restricted to the palisade parenchyma that is in direct contact with the upper epidermis (86% of the total number of feeding events on bean leaves and 77% on *Arabidopsis* leaves, Table 1 and Figures 2B,D). The frequency of feeding events involving multiple cells negatively correlated with the number of dead cells. On bean and *Arabidopsis* leaves respectively,*two* stained cells were observed in 13 and 20% feeding events, and three stained cells were observed in 1 and 3% of feeding events. Thus, when placed on the adaxial epidermis, mites usually damaged a single palisade cell that was immediately below the epidermis. However, the epidermis was not damaged (Table 1 and Figures 2B,D).

**Table 1:** **Distribution of trypan blue stained cells within the bean and ***Arabidopsis*** leaf tissues resulting from ***T. urticae*** feeding for 10 min**.

**Leaf surface exposed**	***Arabidopsis***		**Bean**	
	**Upper**	**Lower**	**Upper**	**Lower**
**Number of damaged cells (%)**				
1	77	69	86	76
2	20	29	13	23
3	3	2	1	1
**Cell type (%)**				
Epidermis	0	0	0	0
Palisade mesophyll (only)	73	16	85	21
Spongy mesophyll (only)	8	64	8	62
Palisade and spongy mesophyll	19	20	7	17
**Samples analyzed (*****n*****)**	95	119	221	207

When mites fed from the abaxial epidermis (where the TSSM preferentially feeds), the damage most frequently occurred in the layer immediately adjacent to the lower epidermis, the spongy parenchyma, in both bean and *Arabidopsis* leaves (76% of feeding events on bean and 69% on *Arabidopsis* involved a single cell in the spongy layer, Table 1 and Figures 2C,E). Feeding that resulted in staining of two or three cells was observed in 24 and 31% of cases in bean and *Arabidopsis* leaves, respectively. Thus, spider mite feeding events (restricted to a 10-min period) most frequently resulted in the trypan blue staining of a *single* mesophyll cell that was adjacent to the epidermal layer that mites stand on, regardless of the plant host. This pattern suggests that mites: (a) do not have a preference for the cell type within the mesophyll, and (b) feed on internal leaf tissue cells without an apparent disturbance of the epidermal layer.

### *Tetranychus urticae* feeding

**Figure 3:**
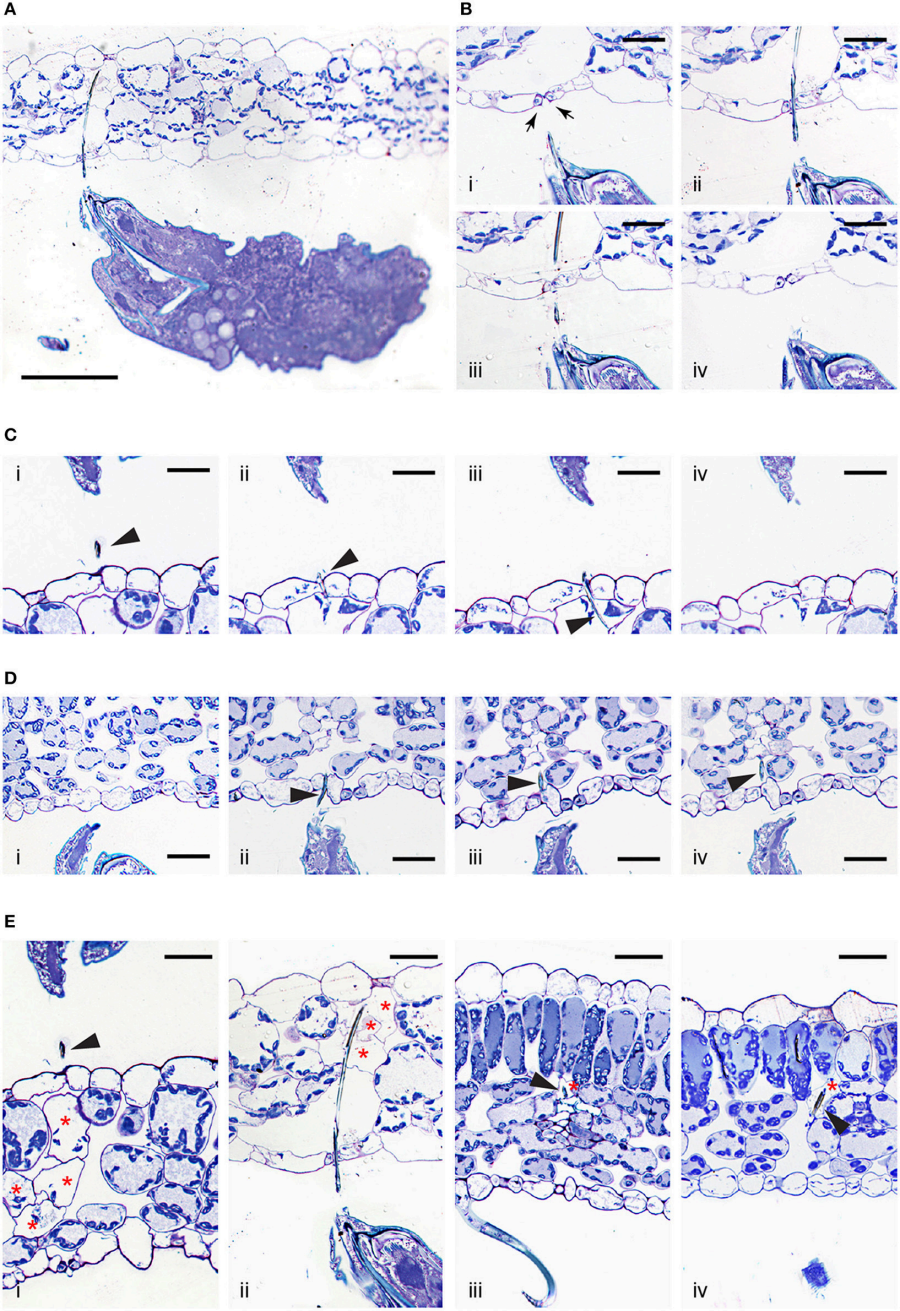
**Interface between ***T. urticae*** and plant tissues during feeding**. **(A)**, A longitudinal cross section of mite feeding from the abaxial side of the *Arabidopsis* leaf. **(B,C)**, Serial sections (1μm apart) of stylet penetration through epidermal cellular layers of *Arabidopsis* leaves while mites fed from the abaxial side (in **B**) and adaxial side (in **C**). Guard cells were labeled with arrows in **(B)**. **(D)**, *T. urticae* stylet penetration while feeding from the abaxial side of bean leaf. **(E)**, Plant cell damage associated with spider mite feeding. Longitudinal cross sections of *Arabidopsis* (i and ii) and bean (iii and iv) leaves showing cells (marked with asterisks) that were in direct contact with mite stylet. Stylet is marked with arrowhead throughout. Scale bars: 100μm in **(A)**, 25μm in **(B–E)**.

A lack of trypan blue staining within the epidermal cellular layers and the observed cell death of mesophyll cells resulting from mite feeding raises the question of stylet penetration through the plant tissue. Given these observations, at least two possible stylet paths are conceivable. First, the stylet can transverses epidermal cells, however, since the stylet is only 2μm in diameter, the resulting damage may not affect cell viability. Consequently, epidermal cells remain colorless upon trypan blue staining. Alternatively, the stylet does not penetrate, but transverses the epidermal layer in between cells. Given that mites retract their stylet upon disturbance, in order to distinguish between these possibilities, we first had to develop a tissue preparation method that supports histological analysis of plant tissues with mites (and their stylets) in a feeding position (see Material and Methods). Longitudinal serial sections of feeding mites, recovered with their stylets within leaf tissues, are shown in Figures 3A–E. These histological sections are oriented with the leaf adaxial (upper) epidermis toward the top and the mite's prosoma (anterior end) toward the left. The epidermal pavement cells, which lack chloroplasts, appear empty in cross sections of glutaraldehyde-fixed and toluidine blue-stained preparations. Interspersed among these cells are stomata, natural openings at the leaf epidermis. Stomata can be recognized on the longitudinal leaf cross sections by the presence of guard cells (marked with arrows in Figure 3B), which are smaller in size relative to the pavement cells, contain chloroplasts, stain blue in our preparations, and are positioned above the substomatal cavity. Cells within the mesophyll layer contain chloroplasts that can be seen as blue circles at the cellular periphery in our toluidine-blue stained leaf cross sections (Figure 3).

A low magnification view of a mite feeding on an *Arabidopsis* leaf from the abaxial epidermis is shown in Figure 3A. In this particular case, the stylet penetrates the epidermal cellular layer through the stomatal opening (see Figures 3Bi–iv). Micron-thick serial sections reveal stylet in two adjacent sections that is consistent with stylet's estimated diameter of 2μm. The stylet transverses the epidermis in between the two guard cells that remain intact (see Figures 3Bii,iii), indicating that the stylet uses a stomatal opening to reach the leaf mesophyll. An examination of additional independent feeding events demonstrates that mites do not always use stomata to insert their stylets into the leaf. Serial sections in Figure 3C show stylet penetration during spider mite feeding from the adaxial epidermis of an *Arabidopsis* leaf. The plane of sectioning is not parallel to the stylet, resulting in the presence of stylet segments (marked by arrowheads) in consecutive sections. Importantly, the stylet crosses the epidermis in between the two pavement cells (Figures 3Cii,iii). Similarly, stylet penetration into the bean leaf upon mite feeding from the abaxial epidermis is shown in serial sections, Figure 3D. In this instance, the penetration occurs between pavement cells that are adjacent to stomata, indicating that mites do not have a preference for transversing the leaf epidermis by inserting their stylet through the stomatal opening. Thus, our histological analysis indicates that stylet penetration occurs either through the stomatal opening (between guard cells) or between the pavement epidermal cells, leaving the epidermal cells intact. This stylet penetration path is consistent with the observed lack of cell death and trypan blue staining in the epidermis.

Histological analysis of independent feeding events also allowed us to visualize for the first time, the appearance of cells that were direct targets of stylet penetration. While intact mesophyll cells have chloroplasts at the cell periphery that are stained dark with toluidine blue and a central vacuole that stains light blue, cells in contact with the stylet are either completely empty, or their cellular contents appeared condensed and partially removed (Figure 3E, see cells marked with asterisks). As a single trypan blue-stained mesophyll cell was the most frequently associated with mite feeding, sections shown in Figures 3Eiii,iv identify individual cells in which the stylet path terminated (labeled with the arrow head) and whose contents have been removed, resulting in empty plasmolysed cells. In other sections, multiple cells that were on the stylet path showed extensive damage (Figures 3Ei,ii) correlating with the identification of multiple cells that were stained with trypan blue. A stylet that transversed the stomatal cavity (Figures 3A,B) penetrated cells toward the adaxial side of the leaf. These cells lacked chloroplasts and were collapsed (Figure 3Eii). In addition, Figure 3Ei shows damage to multiple mesophyll cells whose contents were still partially present but were coagulated. Therefore, mite stylet pierces the leaf epidermis in between cells without damaging them to reach mesophyll layer. The stylet either passes between two pavement cells, or through the stomatal opening created by the two guard cells. Spider mite feeding results in the removal of the content of a single or limited number of mesophyll cells that were on stylet path. In some cases, the cell content can be seen, but is coagulated. The mesophyll cells surrounding damaged cells remained intact with unperturbed internal organization.

## Discussion

### Mechanism of TSSM feeding

The reconstitution of complex relationships between mites and their hosts requires an understanding of mite feeding and its impact on plant integrity. In this study we identified several key features of *T. urticae* feeding: first, we showed that stylet penetrates the leaf either in between epidermis pavement cells or through a stomatal opening, without damaging the epidermal cellular layer (Figures 2, 3); second, mites feed from mesophyll cells without a preference for cell type within this layer (Figure 2 and Table 1); third, the similarity of mite feeding on bean (preferred host) and *Arabidopsis* (a non-preferred host for the strain used in this study) indicates that mite feeding pattern is not affected by the plant host preference (Figure 2 and Table 1); fourth, the duration of a single mite feeding event is longer than previously estimated, ranging from several minutes to more than half an hour (Figure 1 and **Supplemental Movies**); fifth, chlorotic spots that form on damaged leaves are not an immediate consequence of mite feeding (Figure 1).

The leaf epidermis, with its cuticular depositions, is one of the constitutive defenses developed by plants to deter pathogen infection and herbivory. However, stomatal openings disrupt epidermal confluence and are used by most microorganisms and some arthropod pests to access the inner leaf cellular layers (Melotto et al., 2008). Some Tetranychid and Tenuipalpidae mites exclusively target stomatal openings for stylet penetration, e.g., stomatal stylet penetration has been proposed for *Tetranychus lintearius*, which feeds on gorse, *Ulex europaeus* (Marriott et al., 2013) and was demonstrated for *Raoiella* mites on wide range of plant hosts (Beard et al., 2012). Exclusive utilization of stoma as an entry point for the stylet of these mites may arise due to the thick cuticle at the epidermis of host plants, which may present an impermeable physical barrier to stylet penetration. Our analyses showed that *T. urticae* transverse the epidermis either through a junction between epidermal pavement cells or through stomata (Figure 3). Given the limited number of independent penetration events available for the histological analysis we cannot exclude the possibility that TSSMs sometimes penetrate the epidermis by puncturing pavement cells, as proposed by Tanigoshi and Davis (1978), and Campbell et al. (1990). However, if that pattern occurs, it is rare (we did not encounter it in any of the 10 complete serial cross sections) and it does not result in disruption of the epidermal cells [we did not observe trypan-blue stained epidermal cells in the analysis of more than 600 independent feeding events (Figure 2 and Table 1)].

Mites exclusively feed from cells within the mesophyll parenchyma of either bean or *Arabidopsis* leaves (Table 1). Most frequently, the disturbed cells were adjacent to the epidermal layer of the leaf surface that mites fed from (Figure 2). However, such a pattern is not a reflection of mite's inability to reach deep into the leaf tissue when feeding. The *T. urticae* stylet is estimated to be up to ~150μm in length (Avery and Briggs, 1968; Ekka, 1969; Sances et al., 1979). As leaf thickness ranges between 100 and 150μm (depending on species and part of the leaf blade), mite stylet can completely transverse a leaf, allowing it to reach either the palisade or spongy mesophyll regardless of the leaf surface mites are on (see Figure 3A for example). Thus, our data suggest that mites have no preference for the mesophyll cell type, but apparently feed from the first parenchyma cell the stylet encounters. While *T. urticae* exclusively feeds from cells within the mesophyll parenchyma, the eriophyoid mites that are smaller in size and have short stylets only feed from the epidermal cells (Gibson, 1974; Krantz and Lindquist, 1979; Rancic et al., 2006; Nahrung and Waugh, 2012). This demonstrates that epidermal cells could be a source of nutrients as well. Thus, the exclusive feeding of *T. urticae* on cells within the mesophyll layer reflects mite's preference for this cell type. The basis for this preference is currently not known.

Mite feeding is not macroscopically visible (Figure 1). It causes limited damage to plant tissues and a feeding event usually results in the death of a single cell (Figure 2 and Table 1). Cells penetrated by the stylet collapse, with chloroplasts that are either completely removed or appear condensed, Figure 3. These changes are consistent with ultrastructural studies of plant damage caused by mite feeding that showed collapsed cells, devoid of any content, or those that contained condensed cellular debris (Tanigoshi and Davis, 1978; Campbell et al., 1990). Importantly, cells surrounding the dead cell remain alive with no apparent damage (Figure 3 and Campbell et al. 1990). In addition, even though the formation of chlorotic spots has been used as a symptom of plant damage caused by mite feeding (Tanigoshi and Davis, 1978; Sances et al., 1979; Albrigo et al., 1981; Campbell et al., 1990; Park and Lee, 2002; Zhurov et al., 2014), they are not an immediate consequence of mite feeding.

Two possible ways were proposed to explain how mites ingest plant nutritive fluids: fluids either surface on the leaf epidermis and mites intake them directly using their buccal cavity (Alberti and Crooker, 1985; Nuzzaci and De Lillo, 1991a), or, mites use their stylet to suck the cell content *in situ* (Summers et al., 1973; Hislop and Jeppson, 1976; Andre and Remacle, 1984). If mites ingest the cell content from leaf surface, then it is unclear how mites can produce sufficient negative pressure to: (1) allow nutritive fluids to pass through the stylet holes that are created in the cell membrane and the cell wall, and are only few microns in diameter; (2) direct movement of nutritive fluids toward the epidermis, especially within the spongy mesophyll, which is greatly enriched in intracellular air space (Figure 2); and, (3) pass the nutritive fluid through the epidermis, which is otherwise non-permeable, to reach the surface. Moreover, contrary to what would be expected if fluid flowed from the damaged site to the leaf surface, we did not observe remains of cellular contents outside of disrupted cells nor in the apoplastic space leading toward the epidermis. We consider this mode of feeding improbable and favor the possibility that mites use their stylet to suck the nutritive fluid *in situ*. This possibility is consistent with our histological analysis showing stylets within cells whose content is either condensed or emptied (Figure 3) (the extremely high frequency of these observations while analyzing independent feeding events indicates that the stylet remains continuously protruded and inserted into the mesophyll cell during feeding). However, if nutritive sap is sucked through the stylet, then some form of pre-oral digestion likely takes place within the feeding cell. Pre-oral digestion will facilitate consumption of cellular organelles whose size exceeds the stylet diameter (e.g., chloroplasts are several microns in diameter while the stylet tube is about 2μm) and will reduce the viscosity of the cellular content. Digestive enzymes aiding the liquefaction of cellular content could originate from the mite's salivary secretions or from the plant cell itself. Predatory mites inject hydrolytic enzymes into their prey's body to liquefy its content (Cohen, 1995). Thus, it is conceivable that phytophagous mites use a similar mechanism to liquefy the content of the plant cell that they feed on. Recently, serine proteases, deoxygenates and lipocalins were predicted to be part of the mite secretome (Villarroel et al., 2016), supporting the possibility that mites inject hydrolytic enzymes into the plant cell to facilitate nutrient acquisition. Alternatively, the tonoplast likely collapses upon stylet penetration, causing the release of vacuolar hydrolytic enzymes into the cytosol, leading to degradation of cellular structures in a manner similar to the vacuole-induced cell death associated with plant defenses against viruses and microbes (Hara-Nishimura and Hatsugai, 2011). The long duration of individual mite feeding events (Figure 1 and **Supplemental Movies**) supports the possibility of a complex process being required to prepare the cell contents for consumption.

### Mechanism of TSSM feeding: comparison with other cell-content feeding herbivores and implications for the host plant defenses

**Figure 4:**
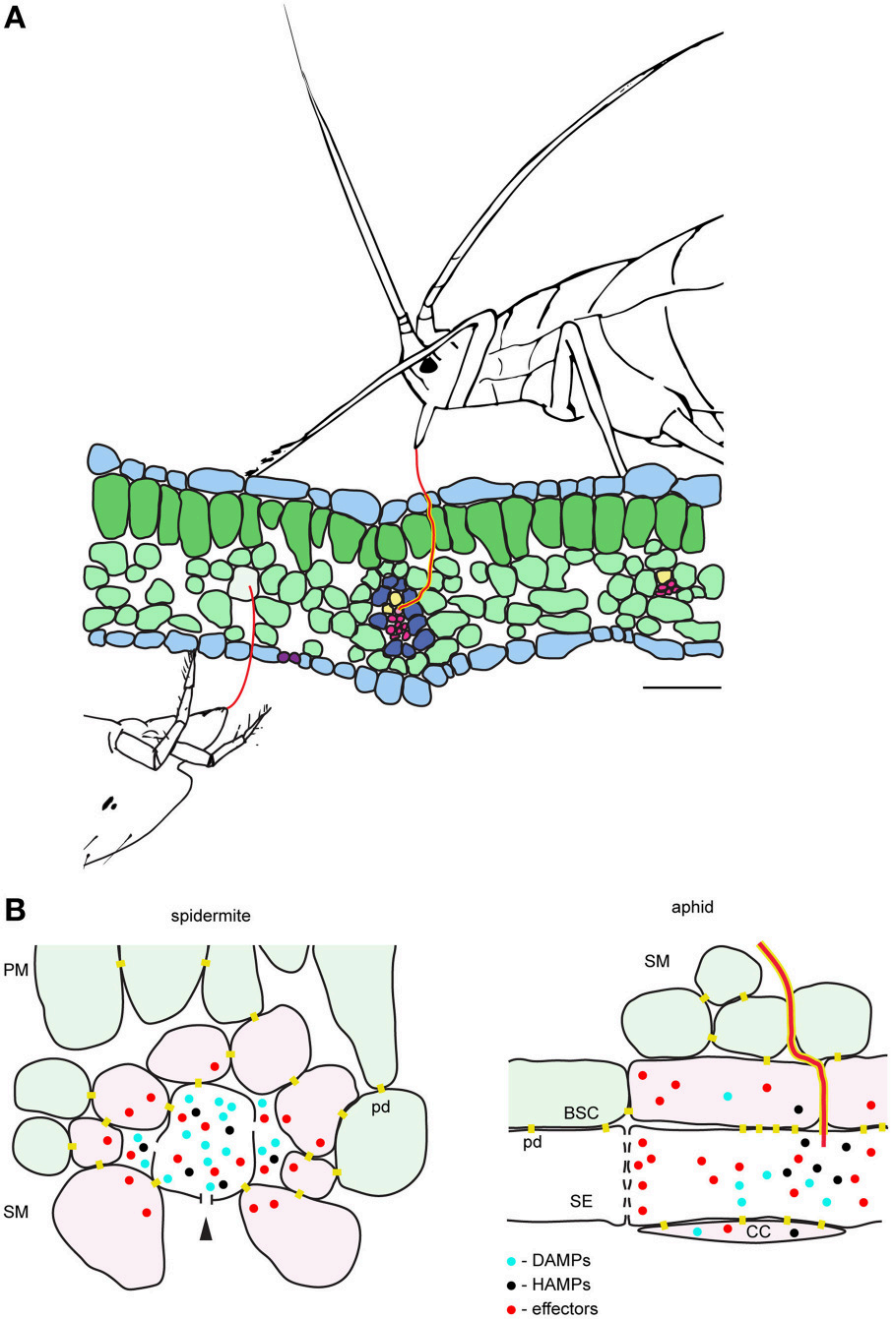
**Model of the interactions between the plant and the cell-content feeding herbivores—the two-spotted spider mite and the aphid**. **(A)**, Schematic of leaf cross-section and feeding herbivores. Both TSSM and aphid use stylets (red) to penetrate the leaf without disturbing epidermal cells. TSSM feeds from the immediate cell in the mesophyll layer that stylet encounters, while aphid navigates its stylets through mesophyll apoplast to reach sieve element cell (SE). The schematic is drawn to scale; scale bar: 50μm. **(B)**, Close-up diagrams of TSSM and aphid feeding sites. On the left, TSSM feeding results in a cell whose content has been removed. Stylet hole (marked with the arrowhead) is disturbing the unity of the plasma membrane and the cell wall. Damage- and Herbivore-Associated Molecular Patterns (DAMPs and HAMPs) are shown as blue and black dots, while mite effector molecules are shown in red. These molecules are expected within the damaged feeding cell and in the apoplast surrounding it, where they may diffuse. Cells that directly respond to DAMPs and HAMPs trigger local responses and are shown in pink. Model predicts that some TSSM effectors (red dots) targeting the modulation of plant transcriptional response are internalized by these cells. Cells surrounding the feeding site and not directly exposed to DAMPs and HAMPs (light green) mount the systemic response. On the right, aphid stylet (red) is surrounded with a salivary sheet (yellow). It penetrates the sieve element cell (SE) where effectors are delivered. Effectors that modulate plant transcriptional reprogramming diffuse into adjacent companion (CC) and bundle sheath (BSC) cells that are symplastically connected with the enucleated sieve element cell. DAMPs and HAMPs also accumulate within the SE and diffuse into CC and BSC cells. PM, palisade mesophyll; SM, spongy mesophyll; pd, plasmodesmata. Schematic in **(B)** is not drawn to scale.

Phytophagous Hemiptera and Acari are cell-content feeders that evolved stylets as part of their feeding apparatus to puncture cells they feed from. Among this group of herbivores, the mechanism of aphid feeding is the best understood (Will et al., 2013; Jaouannet et al., 2014). Both aphids and TSSM use stylets to penetrate the epidermis between pavement cells and to reach feeding cells that are internal to the leaf tissue. However, despite being cell-content feeders, there are some important differences in feeding strategies of these herbivores. For example, aphids have an exclusive preference to feed from sieve element cells within the phloem. Aphids navigate their stylets through the leaf mesophyll and vascular bundle sheath apoplast and, guided by the cell chemical content, reach the phloem to insert the stylet into a sieve element cell (Hewer et al., 2011). Once in a sieve element cell, aphids use their stylet to suck the nutritive sap that is continuously replenished, keeping the feeding cell alive. In contrast, TSSM feeds from mesophyll cells, without an apparent preference for the cell type (Table 1). In addition, mites feed by ingesting and emptying the content of the feeding cell, resulting in cell death. Thus, TSSM feeding takes place in a different cellular context relative to aphids. A model depicting mite and aphid feeding is shown in Figure 4 and their anticipated consequences on induced plant defenses at feeding sites are discussed below.

The duration of a single TSSM feeding event is measured in minutes (Figure 1). During this time, we envision four processes: stylet penetration into the cell, pre-digestion of the cellular content, liquefaction of nutritive fluids and finally the consumption of nutrients. Thus, the feeding cell has limited time to respond to mite feeding. Early responses, including plasma membrane depolarization (measured in seconds Mousavi et al., 2013), changes in ion fluxes (that occur within a minute Felix et al., 1991) and release of signaling/defense compounds (e.g., reactive oxygen species Miller et al., 2009) are expected to occur, but transcriptional reprogramming within the feeding cell is unlikely. Cell wall and membrane fragments generated as a result of stylet penetration, leakage of digested cell content, activation of mechano/turgor-sensitive channels, and potential presence of spider mite salivary secretions are among some of the potential damage- and herbivory-associated molecular patterns (DAMPs and HAPMs respectively) that can act as elicitors at the feeding site. These elicitors are expected to bind to the receptors at the surface of the intact cells surrounding the feeding cell and to trigger local and systemic defense responses, Figure 4B. Spider mite salivary secretions may also contain effectors, aimed at manipulating plant defenses, that are currently being discovered (Villarroel et al., 2016).

Salivary secretions and effectors have been critical for the evolution of the aphid feeding mechanism. Aphids feed for hours (and even days) from a single sieve element cell that remains alive (Tjallingii, 1995). Such prolonged feeding necessitates interference with host defenses that act locally at the feeding site, including: (1) secretion of salivary deposits that form a protective sheet around the stylet as it penetrates the plant tissue in search of the phloem, and, (2) salivary secretions delivered to the sieve element cell to prevent its occlusion and to keep it alive (Figure 4B; Tjallingii, 2006; Will et al., 2007). Interference with the host-induced transcriptional responses at the feeding site occurs in particular in companion cells that have tight symplastic connections with the enucleated sieve element cell. On the other hand, the importance of mite salivary secretions and the cellular processes that are potential targets of mite effectors are presently unknown. The existence of effectors has been monitored through their ability to modify host-induced defenses. Transcriptional changes associated with manipulated plant defenses identified so far indicate that only a subset of induced responses is attenuated (Kant et al., 2008; Alba et al., 2014; Zhurov et al., 2014; Martel et al., 2015; Wybouw et al., 2015; Diaz-Riquelme et al., 2016). Such a pattern suggests that there is an interaction between the effectors and the intracellular components of the responding plant cells, evoking the internalization of effectors deposited in the apoplast at the feeding site. Even though the targets of mite effectors are not known, it is unlikely that they include interference with defense compounds that act locally, as mites move away from the feeding site. It is thus postulated that interference with a plant's ability to mount an effective systemic response will be of greater benefit to spider mite performance.

In summary, we have described the cellular pattern of spider mite feeding. We showed that mites feed from the content of a single mesophyll cell, resulting in its death. A model based on histological observations predicts the existence of both elicitor and effector molecules in the plant apoplast surrounding the feeding cell. The identification of mite effectors and determination of their interacting plant counterparts, as well as the identification of elicitors associated with mite feeding and their receptors, will ultimately allow mapping spatial and temporal events that are triggered by mite feeding.

## Author contributions

VG and MG conceived and together with ID planned the study. NB and MS performed experimental procedures, and collected data. NB, VZ, MG, and VG performed analysis and wrote the manuscript.

## Funding

This work was supported by the Natural Sciences and Engineering Research Council of Canada (NSERC-DG) grant awarded to VG.

### Conflict of interest statement

The authors declare that the research was conducted in the absence of any commercial or financial relationships that could be construed as a potential conflict of interest.

## Supplementary material

Supplemental Movie S1 **Initiation of ***T. urticae*** feeding event**.

Supplemental Movie S2 **Termination of ***T. urticae*** feeding event**.

Supplemental Movie S3 **Feeding of ***T. urticae*** on bean leaf**. Movie has been accelerated 26 times.
